# Isolation and characterization of Nylanderia fulva virus 1, a positive-sense, single-stranded RNA virus infecting the tawny crazy ant, *Nylanderia fulva*

**DOI:** 10.1016/j.virol.2016.06.014

**Published:** 2016-09

**Authors:** Steven M. Valles, David H. Oi, James J. Becnel, James K. Wetterer, John S. LaPolla, Andrew E. Firth

**Affiliations:** a Center for Medical, Agricultural and Veterinary Entomology, USDA-ARS, 1600 SW 23rd Drive, Gainesville, FL 32608, USA; b Wilkes Honors College, Florida Atlantic University, 5353 Parkside Drive, Jupiter, FL 33458, USA; c Department of Biological Sciences, Towson University, 8000 York Road, Towson, MD 21252, USA; d Department of Pathology, University of Cambridge, Cambridge CB2 1QP, United Kingdom

**Keywords:** *Nylanderia fulva*, Tawny crazy ant, RNA virus, Genome sequence, Insect picorna-like virus

## Abstract

We report the discovery of *Nylanderia fulva virus 1* (NfV-1), the first virus identified and characterized from the ant, *Nylanderia fulva*. The NfV-1 genome (**GenBank accession KX024775**) is 10,881 nucleotides in length, encoding one large open reading frame (ORF). Helicase, protease, RNA-dependent RNA polymerase, and jelly-roll capsid protein domains were recognized within the polyprotein. Phylogenetic analysis placed NfV-1 in an unclassified clade of viruses. Electron microscopic examination of negatively stained samples revealed particles with icosahedral symmetry with a diameter of 28.7±1.1 nm. The virus was detected by RT-PCR in larval, pupal, worker and queen developmental stages. However, the replicative strand of NfV-1 was only detected in larvae. Vertical transmission did not appear to occur, but horizontal transmission was facile. The inter-colonial field prevalence of NfV-1 was 52±35% with some local infections reaching 100%. NfV-1 was not detected in limited samples of other *Nylanderia* species or closely related ant species.

## Highlights

• A new positive-strand RNA virus was discovered in the ant, *Nylanderia fulva.*
• The Nylanderia fulva virus 1 genome was comprised of 10,881 nucleotides.
• NfV-1 was detected in larval, pupal, queen and worker ants, but not eggs.
• Replication of NfV-1 appeared to be limited to the larval stage.

## Graphical abstract

**Figure:**
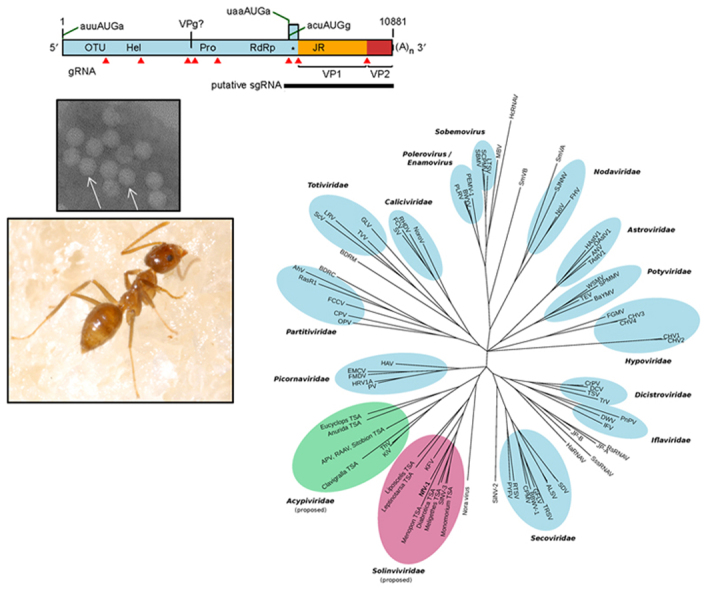

## 1 Introduction

Tawny crazy ant, *Nylanderia fulva* (Mayr), often misidentified as *Paratrechina pubens* and *Nylanderia* nr. *pubens* (Gotzek et al., 2012, Kallal and Lapolla, 2012), was introduced into the United States near Houston, Texas, sometime around 2002 (Meyers and Gold, 2008). A native of South America, this ant has spread rapidly throughout the southeastern U.S. and the Caribbean, and become an acute pest that produces exceedingly large populations that are difficult to control (Deyrup et al., 2000, MacGown and Layton, 2010, Meyers, 2008, Wetterer and Keularts, 2008). Studies have revealed that unique chemical defensive behaviors and venom provide a competitive advantage to tawny crazy ant facilitating its invasive capacity (LeBrun et al., 2014, Zhang et al., 2015). Control efforts are currently limited to chemical insecticides. However, sustainable control of this ant pest will likely require the use of natural enemies. Unfortunately, the only known pathogen of tawny crazy ant is *Myrmecomorba nylanderiae*, a microsporidium that has been identified in the U.S. population (Plowes et al., 2015).

In 2012, we employed a metatranscriptomics—next generation sequencing approach to facilitate discovery of potential viral natural enemies of tawny crazy ant (Valles et al., 2012a). From this work, three short sequence fragments were identified that exhibited strong identity with positive-sense, single-stranded RNA viruses. Completing this investigation here, we used these previously identified sequences as templates to obtain the genome sequence of a new virus, *Nylanderia fulva virus 1* (NfV-1). We describe the genome organization and phylogeny of NfV-1 among related RNA viruses and characterize the virus infection in its host, tawny crazy ant, *N. fulva*.

## 2 Results

### 2.1 Molecular characterization

**Fig. 1:**
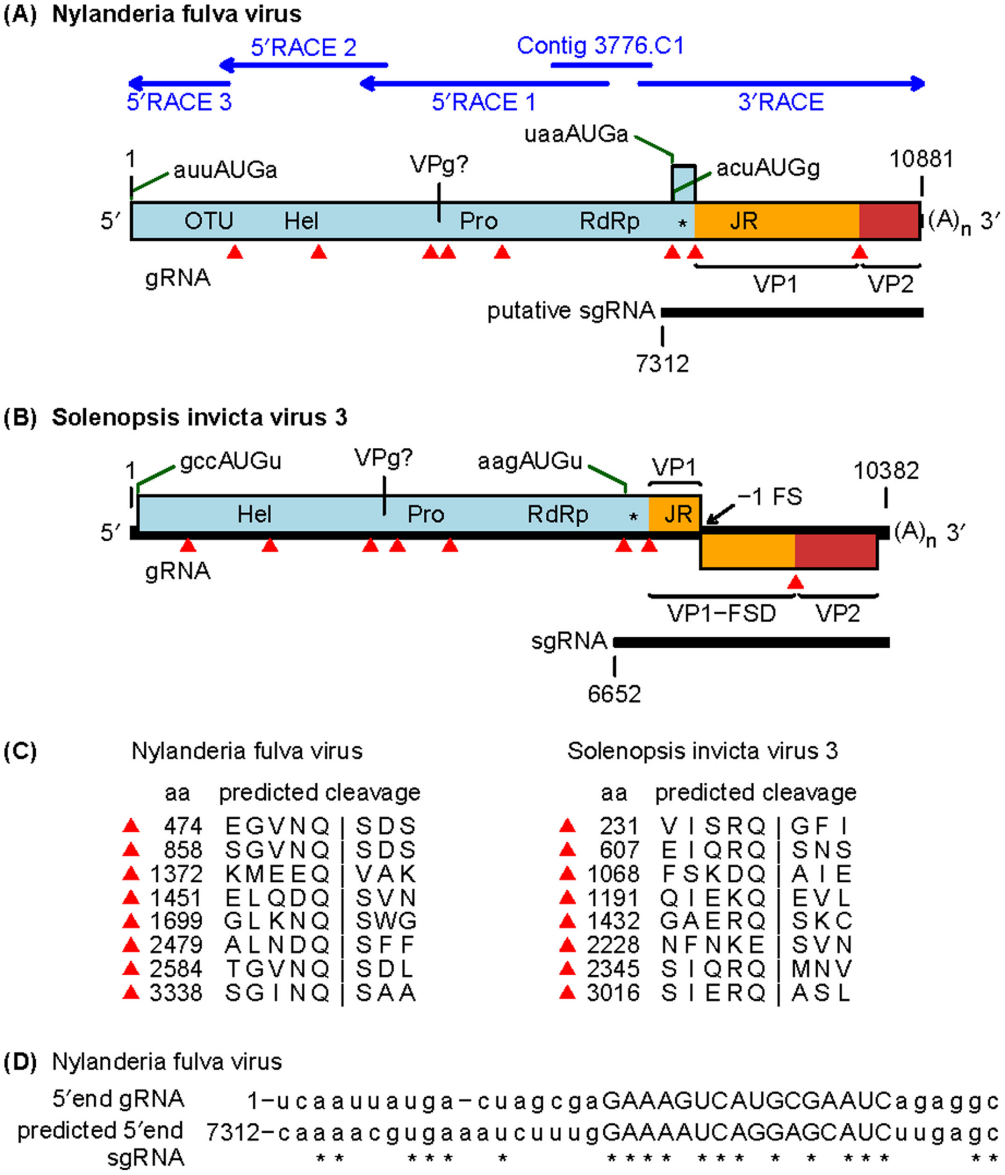
(A) NfV-1 genome organization and method of acquisition. The upper blue arrows represent the cloning strategy for acquiring the NfV-1 genome. Contig 3776.C1 was used as template for initial 5′ and 3′ RACE reactions. Positions of picorna-like helicase (Hel), protease (Pro) and RNA-dependent RNA polymerase (RdRp) protein domains are shown within the polyprotein ORF. Predicted ovarian tumor (OTU), picorna/calici-like jelly-roll fold capsid protein (JR) and dsRNA binding protein (*) domains are also indicated. Potential virus protease cleavage sites are indicated with red triangles. The sequence upstream of Pro is likely to correspond to VPg. A putative sgRNA is indicated. Initiation at the first AUG (poor context) on the sgRNA would lead to translation of a short overlapping ORF while initiation at the second AUG (strong context) would lead to translation of the dsRBP domain and capsid proteins. VP1 (orange) and VP2 (red) are annotated based on analogy with the mapped structural proteins of SINV-3. (B) SINV-3 genome organization and potential cleavage sites. (C) Sequences at the potential cleavage sites in NfV-1 and SINV-3. (D) Comparison of the 5′ terminal sequences of the NfV-1 gRNA and putative sgRNA.

The genome of *Nylanderia fulva virus 1* (NfV-1) was constructed by compiling sequences acquired from a series of three successive 5′ RACE reactions, a 3′ RACE reaction, and contiguous sequence 3776.C1 identified previously from the transcriptome of the ant (Fig. 1(A), Table 1; Valles et al., 2012a). Two other contiguous sequences identified from the earlier study (i.e., 13287.C1 and 8702.C1) were also found to be part of the NfV-1 genome and not from unique viruses. The NfV-1 genome was found to be 10,881 nucleotides in length, excluding the poly(A) tail present on the 3′ end (**Genbank accession KX024775**). The NfV-1 genome sequence contained 58% adenine/uracil, and 42% guanine/cytosine. The genome contains a single large open reading frame (ORF) (Fig. 1(A)). The ORF commences at the first canonical (AUG) start codon, present at nucleotide position 7, ends at a UGA stop codon at nucleotide 10,849, and encodes a predicted polyprotein of 407,455 Da (3614 amino acids). No large ORFs were found in the reverse orientation. The 5′ and 3′ UTRs comprise 6 and 33 nucleotides, respectively. No genome amplification occurred without reverse transcription, consistent with NfV-1 being an RNA virus. The most significant matches from blastp analysis (Altschul et al., 1997) of the polyprotein were to Solenopsis invicta virus 3 (SINV-3) and Kelp fly virus (KFV) with corresponding identities of 26% (65% coverage) and 34% (37% coverage), respectively, while more distant matches clustered in the picornavirus-like “superorder.” Analysis with blastp and HHpred (Söding et al., 2005) identified helicase (Hel), protease (Pro) and RNA-dependent RNA polymerase (RdRp) domains in the N-terminal two thirds of the polyprotein (Fig. 1(A)). These domains contained characteristic motifs for a superfamily III helicase, 3C-like chymotrypsin-related cysteine protease, and a superfamily I RdRp, respectively (Koonin and Dolja, 1993), indicating that NfV-1 is a positive-sense single-stranded RNA virus in the picornavirus-like superorder (Koonin et al., 2008). Given the picornavirales/calicivirus-like Hel-Pro-RdRp arrangement, it is likely that NfV-1 also encodes a VPg (viral protein of the genome) between Hel and Pro. Inspection of NfV-1, SINV-3, KFV and two related sequences (GBSB01003728, LA857567; see below), revealed a short region immediately upstream of Pro, containing many Lys/Arg and Asp/Glu residues, reminiscent of calicivirus VPg proteins (Goodfellow, 2011). In SINV-3 and LA857567, this region contained near identical repeats, two copies of QRKGEKKIKK[V/I]TNYDSDGVQP in SINV-3 and two copies of GDRK[K/T]K[TNF/QKY]VDSDGVQPQ in LA857567 (suggestive of a repeated binding and/or linkage site) while all five sequences contained one or more copies of a [E/D]S[E/D] motif. We suggest that this region may correspond to VPg (Fig. 1(A) and (B)).

Application of HHpred to the NfV-1 polyprotein sequence also revealed an Ovarian Tumor (OTU) domain upstream of Hel, and a dsRNA-binding protein (dsRBP; “*” in Fig. 1(A)) domain and a jelly-roll (JR) capsid protein domain both downstream of RdRp (Fig. 1(A)). Thus, NfV-1 has a genome organization similar to SINV-3 except that SINV-3 appears to lack the OTU domain, and has a ribosomal frameshift site downstream of the JR domain whereas in NfV-1 there is no frameshift (Fig. 1(B)). Potential 3C-like protease cleavage sites were predicted based on the location of predicted protein domains, alignment between NfV-1, SINV-3, KFV and two related sequences (GBSB01003728, LA857567; see below), and sequence homology between different sites within a species (Fig. 1(A)–(C)); it should be stressed that some predictions, particularly those that deviate from a “consensus” sequence, were uncertain.

In SINV-3, the capsid proteins VP1 (comprising the JR domain), VP1-FSD (VP1 with a “Frame Shift Domain” appended, via ribosomal frameshifting) and VP2 (encoded downstream of FSD) can be expressed from the genomic RNA (gRNA); however, a subgenomic RNA (sgRNA) is also produced during virus infection from which only the dsRBP and capsid proteins can be expressed (Fig. 1(B)) (Valles et al., 2014a). VP1 has been hypothesized to form the virion shell, with FSD (present at sub-stoichiometric levels) forming projections; VP2 is also associated with the virion at sub-stoichiometric levels while dsRBP has been hypothesized to be a viral suppressor of RNA interference (RNAi) (Valles et al., 2014a). A similar genome organization is also presented by Acyrthosiphon pisum virus (APV). Due to the similarity between NfV-1 and SINV-3, it is likely that NfV-1 also produces a sgRNA encoding the dsRBP and capsid proteins. A potential sgRNA start site was predicted (Fig. 1(D)) by analogy with SINV-3 (Valles et al., 2014a).

### 2.2 Phylogenetic and comparative analysis

**Fig. 2:**
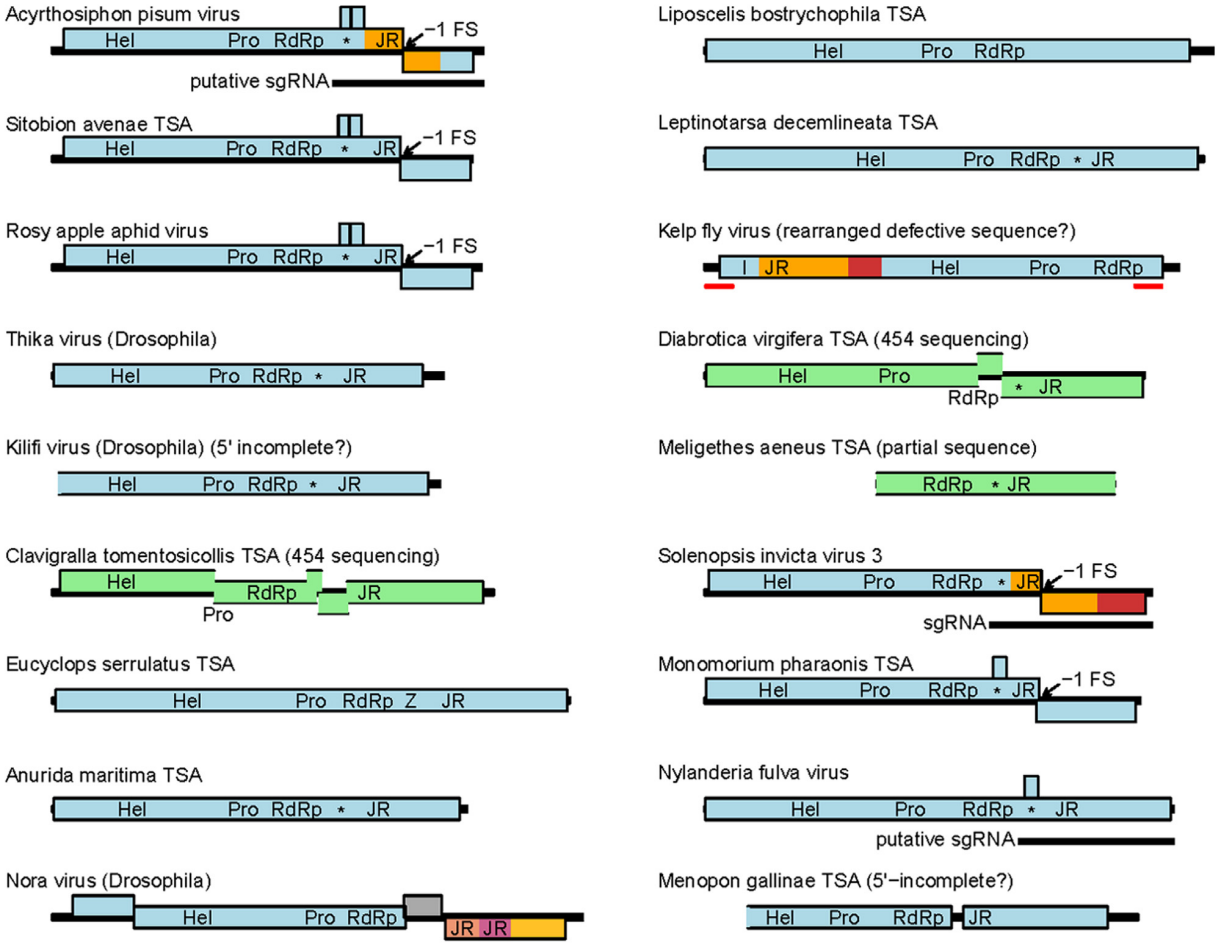
Genome maps of APV, SINV-3, NfV-1, related viruses, and TSA sequences likely to represent additional related viruses. Nora virus, the next most closely related virus (by RdRp-based phylogeny), is also shown for comparison. Sequences assembled from Roche 454 sequence data are shown in pale green with breaks in the polyprotein ORF presumed to have resulted from frame-shift sequencing errors indicated. (The Sitobion TSA also derives from 454 sequencing but high similarity to APV permitted unambiguous removal of three frame-shift errors.) Likely partial sequences are also indicated by un-closed rectangles, although it is possible that additional sequences are incomplete and therefore that some ORFs may be 5′-truncated. Red lines in KFV indicate a 638-nt sequence duplication that may indicate a rearranged defective sequence. Experimentally mapped capsid proteins for APV (van der Wilk et al., 1997), KFV (Hartley et al., 2005), Nora virus (Ekström et al., 2011), and SINV-3 (Valles et al., 2014a) are indicated in colors other than blue and green. Ribosomal −1 frameshift sites are indicated. Protein domains identified by conserved characteristic motifs (Hel – superfamily III helicase; Pro – 3C-like protease; RdRp – superfamily I RdRp) (Koonin et al., 2008) or HHpred (* – dsRNA binding domain; Z – zinc-finger domain; JR – picorna/calici-like jelly-roll capsid domain), as well as the baculoviral inhibitor of apoptosis repeat domain (I) in KFV identified by Hartley et al. (2005), are indicated. The SINV-3 sgRNA and corresponding putative sgRNAs for APV and NfV-1 are indicated; however we expect that all these viruses produce a similarly located sgRNA for capsid protein expression.

Sequences similar to NfV-1, SINV-3 and APV were identified in the NCBI non-redundant nucleotide (nr/nt) and Transcriptome Shotgun Assembly (TSA) databases using tblastn. A total of 17 sequences with full or substantial genome coverage were selected for further analysis (Supplementary Table 1; Fig. 2). Transcriptome shotgun assembly sequences are typically derived from RNASeq (Illumina or Roche 454 platforms) analysis of cellular organism transcriptomes. The genomes of RNA viruses infecting the target organisms are often inadvertently sequenced and assembled as part of the transcriptome, and can be recognized by similarity to known viral sequences. However, as the TSA sequences have not been shown to correspond to infectious viruses, it is possible that some derive from defective viral sequences (such as transcribed host-genome-integrated virus-derived fragments; Katzourakis and Gifford, 2010), or have in silico assembly errors. TSA-derived sequences are also often incomplete and, particularly for 454 sequencing, often have frame-shift sequencing errors. Therefore caution is required when using TSA-derived virus sequences for virus taxonomy and comparative genomic analysis.

**Fig. 3:**
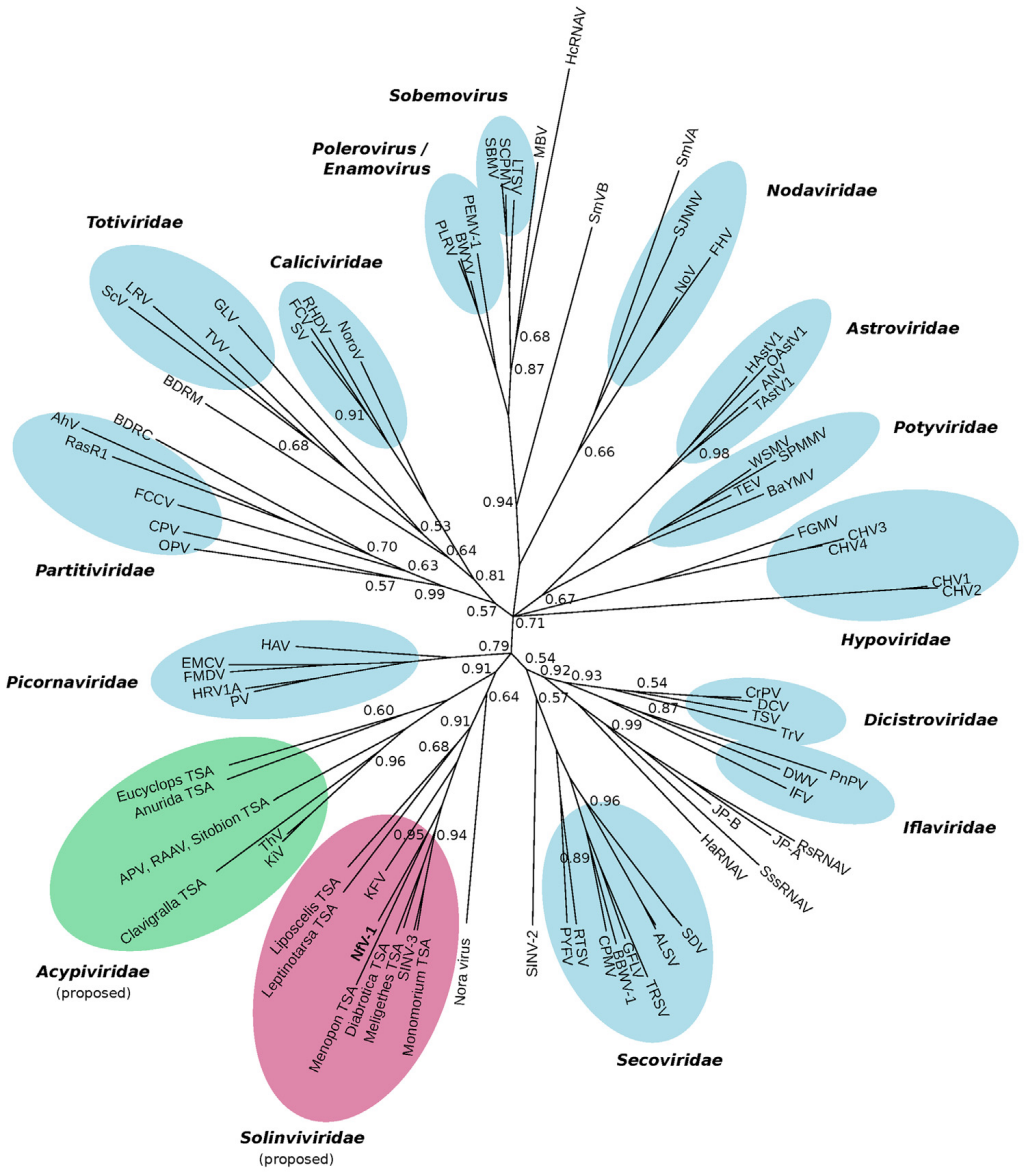
Phylogenetic tree for picorna-like viruses. RdRp amino acid sequences from picorna-like viruses were obtained from Koonin et al. (2008), combined with the equivalent regions from the 17 sequences in Supplementary Table 1, realigned with MUSCLE, and a Bayesian Markov chain Monte Carlo based phylogenetic tree produced. Posterior probabilities are indicated where *p*<1.00. The APV-like and SINV-3/NfV-1-like clades discussed herein are indicated with green and red ellipses, respectively. Within these two clades, posterior probabilities were >0.9 except for the placement of Nora virus within these two clades (*p*=0.64), the placement of the *Liposcelis* and *Leptinotarsa* TSAs within the SINV-3 clade (*p*=0.68), and the placement of the *Eucyclops* and *Anurida* TSAs within the APV clade (*p*=0.60). Abbreviations: AhV, Atkinsonella hypoxylon virus; ALSV, apple latent spherical virus; ANV, avian nephritis virus; APV, Acyrthosiphon pisum virus; BaYMV, barley yellow mosaic virus; BBWV-1, broad bean wilt virus 1; BDRC, Bryopsis cinicola chloroplast dsRNA replicon; BDRM, Bryopsis mitochondria-associated dsRNA; BWYV, beet western yellows virus; CHV, Cryphonectria parasitica hypovirus; CPMV, cowpea mosaic virus; CPV, Cryptosporidium parvum virus; CrPV, cricket paralysis virus; DCV, Drosophila C virus; DWV, deformed wing virus; EMCV, encephalomyocarditis virus; FCCV, Fragaria chiloensis cryptic virus; FCV, feline calicivirus; FGMV, Fusarium graminearum mycovirus; FHV, flock house virus; FMDV, foot-and-mouth disease virus; GFLV, grapevine fanleaf virus; GLV, Giardia lamblia virus; HaRNAV, Heterosigma akashiwo RNA virus; HAstV1, human astrovirus 1; HAV, hepatitis A virus; HcRNAV, Heterocapsa circularisquama RNA virus; HRV1A, human rhinovurus 1A; IFV, infectious flacherie virus; JP-A, marine RNA virus JP-A; JP-B, marine RNA virus JP-B; KFV, kelp fly virus; KiV, Kilifi virus; LRV, leishmania RNA virus 1-1; LTSV, lucerne transient streak virus; MBV, mushroom bacilliform virus; NfV-1, Nylanderia fulva virus 1; NoroV, norovirus; NoV, Nodamura virus; OAstV1, ovine astrovirus 1; OPV, Ophiostoma partitivirus 1; PEMV-1, pea enation mosaic virus 1; PLRV, potato leafroll virus; PnPV, Perina nuda picorna-like virus; PV, poliovirus; PYFV, parsnip yellow fleck virus; RAAV, rosy apple aphid virus; RasR1, Raphanus sativus dsRNA 1; RHDV, rabbit haemorrhagic disease virus; RsRNAV, Rhizosolenia setigera RNA virus; RTSV, rice tungro spherical virus; SBMV, southern bean mosaic virus; SCPMV, southern cowpea mosaic virus; ScV, Saccharomyces cerevisiae virus LA; SDV, satsuma dwarf virus; SINV-2, Solenopsis invicta virus 2; SINV-3, Solenopsis invicta virus 3; SJNNV, striped jack nervous necrosis virus; SmVA, Sclerophtora macrospora virus A; SmVB, Sclerophtora macrospora virus B; SPMMV, sweet potato mild mottle virus; SssRNAV, Schizochytrium single-stranded RNA virus; SV, Sapporo virus; TAstV1, turkey astrovirus 1; TEV, tobacco etch virus; ThV, Thika virus; TRSV, tobacco ringspot virus; TrV, Triatoma virus; TSV, Taura syndrome virus; TVV, Trichomonas vaginalis virus 1; WSMV, wheat streak mosaic virus.

To establish an RdRp-based phylogeny, we extracted the picornavirus-like superorder RdRp alignment provided in supplementary file 2 of Koonin et al. (2008), appended the equivalent region (based on alignment to APV) from the 17 sequences in Supplementary Table 1, and rebuilt the alignment using MUSCLE (Edgar, 2004). We then used MrBayes (Ronquist et al., 2012) to generate a Bayesian Markov chain Monte Carlo based phylogenetic tree (Fig. 3). The analysis revealed that SINV-3-like and APV-like sequences form two distinct clusters which may form the basis of two new virus families (tentative names *Solinviviridae* [type species ***Sol***enopsis ***invi***cta virus 3] and *Acypiviridae* [type species ***Acy***rthosiphon ***pi***sum virus]. NfV-1 clusters within the SINV-3 clade.

Within the APV-clade, three closely related aphid-derived sequences (APV, rosy apple aphid virus and a *Sitobion avenae* [Arthropoda: Insecta: Hemiptera] TSA) have similar genome structure, including conservation of two predicted overlapping genes that might be translated via leaky scanning on the inferred sgRNA (Fig. 2) (Valles et al., 2014a). Thika and Kilifi viruses, both isolated from *Drosophila* species (Arthropoda: Insecta: Diptera) (Webster et al., 2015), lack the ribosomal frameshift site identified in APV (van der Wilk et al., 1997) but instead have monocistronic genomes. Nonetheless they maintain the genome order Hel-Pro-RdRp-dsRBP-JR. Due to the lack of frameshifting, instead of producing the 33K (VP1) and 66K (VP1-FSD) capsid proteins of APV, they would produce just a single JR-containing capsid protein (presumably equivalent to VP1-FSD, giving a 1:1 stoichiometry of shell: projection domains). The *Clavigralla tomentosicollis* (Arthropoda: Insecta: Hemiptera) TSA, derived from 454 sequencing, apparently had a number of frame-shift sequencing errors, but no change in reading frame corresponding to the APV frameshift site. Thus the sequence from *Clavigralla tomentosicollis* is likely to be monocistronic, similar to the Thika and Kilifi virus sequences to which it is most closely related. More distantly related were the TSAs from *Eucyclops serrulatus* (Arthropoda: Maxillopoda: Cyclopoida) and *Anurida maritima* (Arthropoda: Collembola: Poduromorpha). For the *Eucyclops serrulatus* TSA, the characteristic dsRBP domain downstream of RdRp was not found but instead was replaced with a predicted zinc-finger domain.

Within the SINV-3/NfV-1 clade, a TSA sequence from *Monomorium pharaonis* (Arthropoda: Insecta: Hymenoptera) had a very similar genome organization to SINV-3 but also encoded a potential overlapping gene that might be translated from the inferred sgRNA (Fig. 2). A potential overlapping gene is also present at this location in NfV-1 and is the only AUG-initiated ORF in the NfV-1 genome apart from the polyprotein ORF with length >100 codons. The *Diabrotica virgifera* (Arthropoda: Insecta: Coleoptera) TSA, derived from 454 sequencing, had two frame-shift sequencing errors which could be accurately corrected by amino acid alignment to the *Meligethes aeneus* (Arthropoda: Insecta: Coleoptera) TSA sequence, following which both the *Diabrotica virgifera* and partial *Meligethes aeneus* sequences appeared to be monocistronic, but otherwise consistent with the SINV-3 genome architecture. More divergent TSA sequences from *Liposcelis bostrychophila* (Arthropoda: Insecta: Psocoptera) and *Leptinotarsa decemlineata* (Arthropoda: Insecta: Coleoptera) also appeared to be monocistronic but, while the *Leptinotarsa decemlineata* TSA displayed the characteristic gene order Hel-Pro-RdRp-dsRBP-JR, the dsRBP and JR domains could not be identified with HHpred in the *Liposcelis bostrychophila* sequence. It is possible that this sequence represents a “chimeric” virus that has acquired a different C-terminal gene block (perhaps containing novel capsid proteins) or, alternatively, the TSA sequence may be misassembled at the 3′ end. Finally, a TSA sequence from *Menopon gallinae* (Arthropoda: Insecta: Phthiraptera) appeared to be dicistronic. While we could not rule out sequencing/assembly errors being responsible for the “broken” ORF, the dicistronic architecture with Hel-Pro-RdRp encoded in the 5′ ORF and the capsid proteins encoded in the 3′ ORF would be compatible with the production of a sgRNA in viruses of this clade. KFV has a rearranged genome structure and may be misassembled (see discussion in Valles et al. (2014a)).

In contrast, despite similarity in the RdRp region, Nora virus has a quite different genome architecture: ORF2 encodes picorna-like Hel, Pro and RdRp domains, ORF4 encodes three proteolytically cleaved capsid proteins (where the first and likely the second contain JR domains), ORF3 encodes a minor capsid protein, and ORF1 encodes a viral suppressor of RNAi (Fig. 2) (Ekström et al., 2011, van Mierlo et al., 2012). Although the RdRp phylogenetic analysis placed Nora virus within the SINV-3/APV sister clades, the posterior probability for this placement was only 0.64.

### 2.3 Purification and electron microscopy

**Fig. 4:**
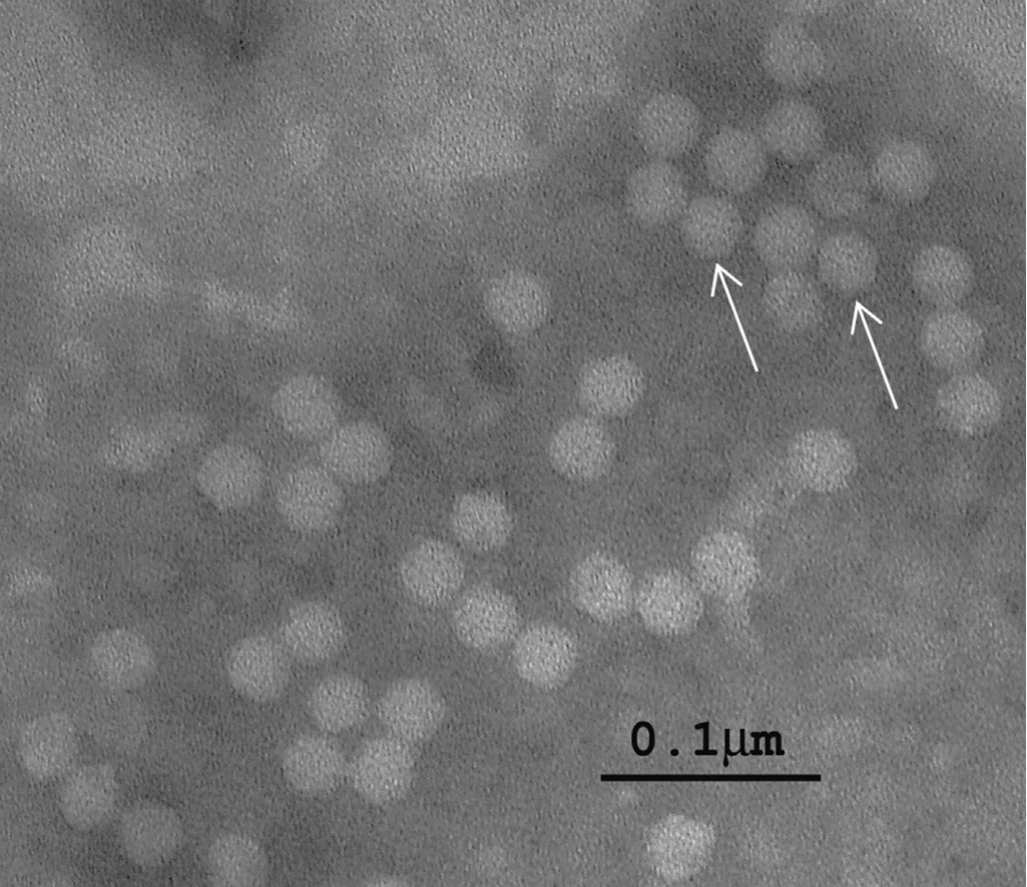
Electron micrograph of a negative stain of Nylanderia fulva virus 1 purified by CsCl isopycnic centrifugation. A group of intact virus particles demonstrating the icosahedral symmetry (arrow) and a surface with numerous small projections (spikes). Mean diameter of virus particles was 28.7±1.1 nm.

Particles purified from NfV-1-infected tawny crazy ants migrated to a density of 1.287±0.012 g/ml CsCl. Electron microscopic examination of negatively stained samples revealed particles with icosahedral symmetry, vaguely visible surface projections (spikes), and a diameter of 28.7±1.1 nm (Fig. 4). RT-PCR of RNA prepared from this purified virus preparation verified that these particles contained the NfV-1 genome. No corresponding particles were observed in samples prepared from uninfected ants.

### 2.4 NfV-1 infection characteristics in the ant host and field prevalence

**Fig. 5:**
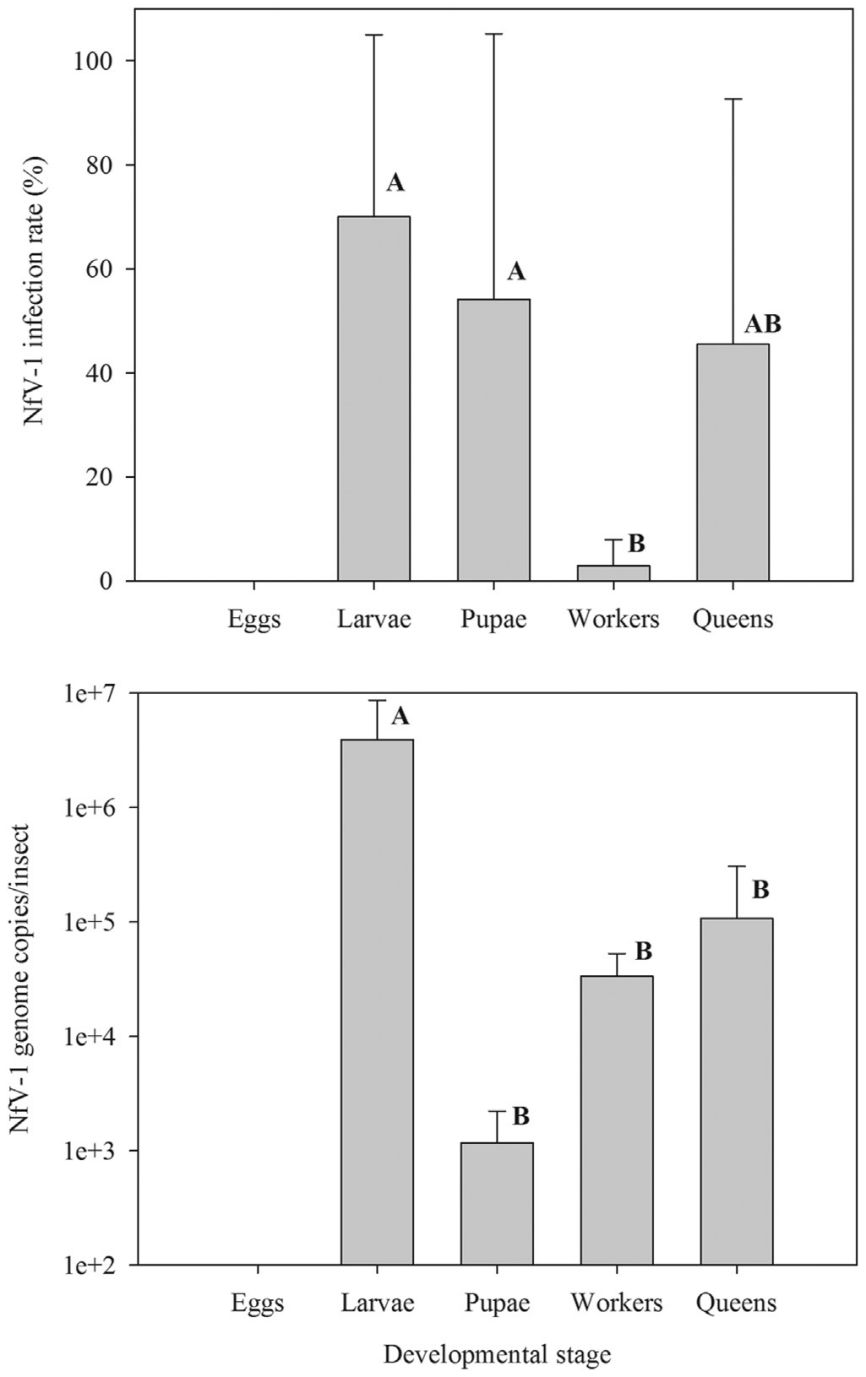
Intra-colonial prevalence (upper) and quantity (lower) of Nylanderia fulva virus 1 in different developmental stages of the host, *Nylanderia fulva*. Colonies were pre-determined to be infected with NfV-1 and then individuals from different life stages were tested to determine the mean prevalence of the virus by RT-PCR. Individuals testing positive by RT-PCR were subsequently evaluated by QPCR.

NfV-1 was detected in all stages of the ant examined except eggs. NfV-1 was regularly detected in workers, larvae, pupae, and queens (Fig. 5). Intra-colonial tests revealed that larvae, pupae and queens had the highest infection rates for the virus. Although NfV-1 was detected in workers, the proportion of workers (2.9%) testing positive for the presence of the virus was significantly (*p*<0.05) lower compared with larvae, pupae, and queens (Fig. 5). NfV-1 titer was also stage dependent. While NfV-1 was detected in larval, pupal, worker, and queen stages, the amount of virus present (based on genome equivalents) was significantly higher in the larval stage (by several orders of magnitude). NfV-1 was not detected in eggs collected from known NfV-1-infected colonies (*n*=4) or collected directly by known NfV-1-infected queens (*n*=5) suggesting that virus is not vertically transmitted.

Among 168 separate colonies of *N. fulva*, the mean inter-colonial prevalence of NfV-1 was 52.4% (Table 2). Local infections were found to be as high as 100%. No apparent relationship was observed between virus prevalence and season as NfV-1 was found during all times of the year. The virus was not detected in limited, archived samples from St. Croix, Virgin Islands, or from 11-different *Nylanderia* species and closely related *Prenolepis* species (Table 2, Table 3).

NfV-1 was transmitted to uninfected colonies by feeding. NfV-1 was detected in *N. fulva* ant colonies after a 3-day pulse exposure to a sucrose solution containing a homogenate of ants infected with NfV-1 after a 10 day incubation period. The virus was actually easily spread because some of the control colonies, maintained in the same room as the NfV-1-treated colonies also became positive for NfV-1 infection. The minus (replicative) strand of NfV-1 was detected in RNA samples collected from larvae, but not workers, indicating replication of the virus, confirming previous experiments (Valles et al., 2012a). No significant differences were observed in the percent change from the initial brood and worker levels at 19 weeks after inoculation between colonies with persistent NfV-1 infections (*n*=3) and uninfected colonies (*n*=5, Table 4). Colonies were considered to have persistent infections when NfV-1 was detected at two sampling points. Two of these colonies were intentionally inoculated, while the other colony was a control colony. Of the uninfected colonies, two control colonies had virus detected only in the second week. Oviposition rates between NfV-1-infected and -uninfected queens did not differ significantly (*t*=1.24; df=16; *p*=0.23). Five infected queens produced a mean (±SE) of 38.4 (±19.9) eggs/24 h, while the uninfected queens (n=13) exhibited a mean oviposition rate of 67.5 (±12.4) eggs/24 h.

## 3 Discussion

Recent emergence and limited scientific inquiry have hampered efforts to control tawny crazy ant. Control methods are limited currently to chemical insecticides, so discovery of natural enemies is sorely needed to develop sustainable control methods. To date, efforts to identify biologically-based control organisms of tawny crazy ant have led to the discovery of *Myrmecamorba nylanderiae*, a microsporidium (Plowes et al., 2015) and putative viral pathogens (Valles et al., 2012a). The putative viruses were identified in silico using a metatranscriptomics—next generation sequencing approach (Valles et al., 2012a). Blast analysis (Altschul et al., 1997) of nucleotide sequences from a gene library collected from nine geographically separated colonies identified 51 unique sequences of putative viral origin. Three of these sequences (13287.C1, 3776.C1, 8702.C1) were shown to be likely from replicating viruses that infect the ant (Valles et al., 2012a). Using sequence 3776.C1 as a starting point, oligonucleotide primers were designed and RACE (3′ and 5′) reactions conducted leading to the discovery of a new virus, *Nylanderia fulva virus 1* (NfV-1). The 10,881 nucleotide RNA genome was monopartite, monocistronic, single-stranded, and possessed a poly(A) tail at the 3′ end of the genome. Sequencing also revealed that the three fragments identified in the earlier study (i.e., 13287.C1, 3776.C1, 8702.C1) were all part of the same virus genome.

Analysis of the NfV-1 genome revealed superfamily III helicase, 3C-like protease, and superfamily I RdRp domains in the order Hel-Pro-RdRp, characteristic of members of the order Picornavirales and family *Caliciviridae*. A single picorna/calici-like jelly-roll capsid domain was predicted downstream of the RdRp domain, making the genome structure more calici-like than picorna-like. The related virus SINV-3 has been shown to produce a sgRNA for capsid protein expression, which is another calici-like feature of these viruses. Phylogenetic analysis of the RdRp amino acid sequence placed NfV-1 in a clade with SINV-3, KFV and several arthropod-derived TSA sequences. APV, rosy apple aphid virus, Thika virus, Kilifi virus and several other arthropod-derived TSA sequences formed a separate sister clade (Fig. 3). In both clades, there existed sequences in which ribosomal frameshifting downstream of the jelly-roll capsid domain would result in sub-stoichiometric levels of the extension domain (“FSD”) and VP2, and sequences in which these domains were fused in-frame with the jelly-roll domain apparently resulting in a 1:1 stoichiometry. Notwithstanding the similarities in genome architecture, there was no evidence based on the RdRp tree that these viruses clustered more closely with caliciviruses than picornaviruses. On the other hand, a previous analysis of the helicase domain, placed APV and KFV closer to caliciviruses than picornaviruses (Koonin et al., 2008). These two clades of “calici-like” arthropod-infecting positive-sense RNA viruses may form the bases of two new families for which we tentatively propose the names *Solinviviridae* (***Sol***enopsis ***invi***cta virus 3 as the type species, NfV-1, and relatives) and *Acypiviridae* (***Acy***rthosiphon ***pi***sum virus as the type species, Thika virus, and relatives).

Unlike caliciviruses, but in common with other insect viruses, most members of the two clades were predicted to encode a viral suppressor of RNAi, generally taking the form of a dsRNA binding domain (“*” in Fig. 2) with the same predicted fold as the 1A suppressor of silencing protein of Drosophila C virus (family *Dicistroviridae*) (van Rij et al., 2006). In the *Eucyclops serrulatus* TSA, the dsRBP domain was not predicted but instead HHpred predicted a zinc-finger domain at the same genomic location (“Z” in Fig. 2). While many zinc-finger domains are involved in DNA binding, others are involved in RNA binding (Hall, 2005); thus this may be an alternative viral suppressor of RNAi. Unusually, NfV-1 contained a predicted OTU domain upstream of the helicase domain. OTU domains have been found in a few diverse RNA viruses, where they can be involved in proteolytic cleavage of viral polyproteins and, via a de-ubiquitination/de-ISGylation activity, inhibition of innate immune signaling (Bailey-Elkin et al., 2014, Frias-Staheli et al., 2007, van Kasteren et al., 2012).

NfV-1 was detected in larval, pupal, worker, and queen stages (Fig. 5). The virus was not detected in eggs indicating that it is not vertically transmitted and is likely acquired by horizontal transmission (intra- and inter-colony). Formal inter-colonial transmission tests and frequent laboratory NfV-1 infections of newly acquired field colonies of tawny crazy ant support this conclusion. However, transmission studies of NfV-1 to uninfected ants were equivocal. While transmission to uninfected tawny crazy ant colonies occurred, a corresponding control colony also acquired the infection. NfV-1-treated and -untreated colonies were housed in the same facility, so cross contamination was a concern. Indeed, the tawny crazy ant insectary was regularly contaminated with NfV-1. Virus-free colonies retrieved from the field and held in separate rearing trays in our facility invariably acquired the infection. In fact, a preliminary transmission study testing an NfV-1 bait formulation resulted in both inoculated and control colonies (*n*=2) becoming infected (D.H.O unpublished data). This same problem was encountered with SINV-3 and its host, the red imported fire ant, *Solenopsis invicta* (Valles and Porter, 2012, Valles et al., 2013, Valles et al., 2014b).

QPCR data of different developmental stages corresponded with the intra-colonial developmental infection rate of NfV-1; the larval stage of tawny crazy ant contained significantly higher quantities of NfV-1 compared with adults (workers and queens) and pupae. The purpose of these experiments was to determine which developmental stages were infected, not necessarily to examine viral pathogenesis, so the NfV-1 titer may actually be higher in different stages depending on the infection time course. The NfV-1 replicative strand was only detected in the larval stage. However, failure to detect the replicative strand in the adult stage does not necessarily suggest that it is not involved in NfV-1 pathogenesis. Indeed, low NfV-1 titer and the absence of the replicative genome strand in worker ants may be attributed to RNA degradation. Worker ants were shown previously to contain a potent RNA degrading substance (Valles et al., 2012b). Localized to the abdomen, liberation of this endogenous substance resulted in severe to complete RNA degradation in preparations from tawny crazy ant workers (Valles et al., 2012b).

Perfunctory host specificity tests suggest that NfV-1 infection may be limited to *N. fulva* according to the phylogeny-based system proposed by Briese (2005). The virus was not detected in congeners or closely related species outside the *Nylanderia* genus (Table 3). However, a more thorough study, including direct challenge with NfV-1, will be required to establish the host specificity.

Use of RNA viruses as a viable method of ant control has been proposed previously (Oi and Valles, 2009, Valles, 2012). SINV-3 has been successfully released into field populations (Florida and California) of *S. invicta* as a classical biological control agent (Valles and Oi, 2014) and has shown promise as a biopesticide (Valles and Porter, 2015, Valles et al., 2013). Despite a lack of overt symptoms among tawny crazy ants infected with NfV-1, one of the infected colonies used from the transmission experiment declined over time exhibiting significant mortality among brood (83%) and workers (76%). However, other colonies seemed to thrive despite NfV-1 infection. Mean oviposition rates of infected queens was 43% lower than uninfected queens, however the sample size of infected queens was small and the reduced rate was not statistically significant. Field populations of *N. fulva* have been observed (DHO, personal observation) and reported to exhibit unexplained extensive reductions and corresponding disappearance from established areas (Wetterer et al., 2014). This type of population cycling is commonly observed in introduced populations trying to establish themselves in a new area (Simberloff and Gibbons, 2004). Many RNA viruses are known to persist as inapparent infections that begin actively replicating when the host experiences some sort of external stress or co-infection with another pathogen (Chen et al., 2012, Christian and Scotti, 1996, Scotti et al., 1981). Thus, NfV-1 alone, or in combination with another pathogen, may be contributing to this boom-bust cycle observed in tawny crazy ant. Obviously, the impact of NfV-1 on tawny crazy ant colonies will have to be examined more closely.

## 4 Materials and methods

### 4.1 Ant collections

*Nylanderia fulva* colonies were collected from field sites in Gainesville (Alachua Co.), near Winter Garden (Lake Co.), Lakewood Ranch (Manatee Co.), and Callahan (Nassau Co.) Florida. Colonies were generally obtained from dead branches or in man-made debris such as beverage cans and nested plant pots. For the field prevalence experiments, workers and larvae were often collected under leaf litter and at the bases of trees or shrubs. Multiple collections within a site were a minimum 30 m apart. Ants were anaesthetized on an ice pack and preserved in 95% ethanol in the field. Ants were identified by morphological examination of workers and male alates using available keys and descriptions (Gotzek et al., 2012, LaPolla et al., 2010). *N. fulva* colony samples also were collected at sites in central and western St. Croix, US Virgin Islands (Wetterer et al., 2014).

### 4.2 Genome sequencing and molecular characterization

Three consecutive 5′ RACE reactions were conducted to obtain the sequence upstream from contig 3776. C1. This sequence fragment was identified as being of viral origin and obtained from the transcriptome of *Nylanderia fulva* ants (Valles et al., 2012a). Using the 5′ RACE system (Invitrogen, Carlsbad, CA), cDNA was synthesized for 50 min at 42 °C with 1–3 µg of total RNA obtained from NfV-infected ants with a gene-specific oligonucleotide primer (GSP; Table 5), the RNA template was degraded with RNase H, and the cDNA purified by S.N.A.P. column (Invitrogen, Carlsbad, CA). The 3′ end of the cDNA was polycytidylated and then amplified by PCR with a nested GSP (3′ end) and an abridged anchor primer (AAP). Gel purified amplicons were ligated into the pCR4-TOPO vector, transformed into TOP10 competent cells (Invitrogen, Carlsbad, CA) and sequenced by the Interdisciplinary Center for Biotechnology Research (University of Florida).

One 3′ RACE reaction was conducted with the GeneRacer kit (Invitrogen) also using contig 3776.C1 as template. cDNA was synthesized from total RNA (1 µg) using an oligo dT primer. The cDNA was amplified by PCR with a GSP (Table 5) and the GeneRacer 3′ primer. Amplicons were cloned and sequenced as described for 5′ RACE.

Once a draft of the genome was acquired, overlapping gene specific oligonucleotide primers were designed and used to amplify the entire genome with a minimum of 5-fold coverage.

### 4.3 Sequence analysis

Domains within virus and TSA inferred amino acid sequences were identified using National Center for Biotechnology Information (NCBI) blastp (Altschul et al., 1997) and HHpred (Söding et al., 2005). Sequences with similarity to NfV-1 were identified using tblastn (Altschul et al., 1997). For producing the RdRp phylogenetic tree, peptide sequences were aligned with MUSCLE (Edgar, 2004), and a maximum likelihood phylogenetic tree was estimated using the Bayesian Markov chain Monte Carlo method as implemented in MrBayes version 3.2.3 (Ronquist et al., 2012) sampling across the default set of fixed amino acid rate matrices, with 5 million generations, discarding the first 25% as burn-in.

### 4.4 Virus detection, purification, and electron microscopy

Reverse transcription polymerase chain reaction (RT-PCR) was used to identify NfV-1-infected *Nylanderia fulva* ants, which targeted a portion of the virus genome. *N. fulva* colonies were collected from various locations in Florida, returned to the laboratory, and RNA was extracted from 10 to 20 ants from a colony using Trizol reagent according to the manufacturer׳s directions. cDNA was synthesized with oligonucleotide primer, p1168 (Table 5), and PCR conducted with oligonucleotide primers, p1167 and p1168. Samples were considered positive for the virus when a visible amplicon (358 nucleotides) was present after separation on a 1.2% agarose gel stained with SYBR Safe. PCR was conducted under the following optimized temperature regime: 1 cycle at 94 °C for 2 min, 35 cycles of 94 °C for 15 s, 61 °C for 15 s, 68 °C for 30 s, followed by a final elongation step of 68 °C for 5 min.

NfV-1 was purified by discontinuous and isopycnic centrifugation. A mixture of worker and larval stages (12 g) were homogenized in 120 ml of NT buffer (10 mM Tris–HCl, pH 7.4, 100 mM NaCl) in a Waring blender on high speed for 2 min. The mixture was filtered through 8 layers of cheesecloth and then extracted with an equal volume of chloroform for 10 min with constant shaking. The mixture was centrifuged for 5 min at 5000*g* and the supernatant collected by pipette. The supernatant was layered onto a discontinuous CsCl gradient (1.2 g/ml and 1.5 g/ml), which was centrifuged at 190,000*g* for 2 h in a Ti50.1 rotor. A whitish band visible near the interface was removed and brought to a density of 1.3 g/ml CsCl. This sample was then centrifuged at 330,000*g* for 18 h in a Ti70.1 rotor. A faint opaque band at 1.287±0.012 g/ml was collected. Approximately 10 μl of the gradient purified viral suspension was placed onto a formvar coated grid for 5 min and the excess removed. A 2% (w/v) aqueous phosphotungstic acid (PTA) adjusted to pH 7.5 with 1 N NaOH was applied (10 μl) to the grid for 2 min, the excess was removed and allowed to air dry. The negatively stained specimens were viewed and viral particles photographed with a Hitachi H-600 transmission electron microscope (Hitachi, Pleasanton, CA) at an accelerating voltage of 100 kV.

### 4.5 NfV-1 infection characteristics in the ant host

The NfV-1 intra-colonial infection rate was determined in individual larvae, workers, queens, and pupae of known virus-infected colonies. Total RNA was isolated from individual ants and RT-PCR was conducted as described above using oligonucleotide primers p1167 and p1168. Individuals from 14 different colonies were examined (*n*=87 larvae, 25 pupae, 24 queens, 46 workers). Presence of NfV-1 was also evaluated in egg clusters. RNA was isolated and RT-PCR conducted.

The NfV-1 inter-colonial infection rate was also determined by examining *Nylanderia fulva* colonies collected from different locations in central Florida and St. Croix, Virgin Islands. RNA was isolated from ten ants of various stages (depending on availability) from colonies directly in the field. RT-PCR was subsequently conducted to detect the virus as described.

Virus quantification was conducted by QPCR on an ABI PRISM 7000 Sequence Detection System interfaced to the ABI prism 7000 SDS software (Applied Biosystems, Foster City, CA) in a 25 μl reaction volume. The reaction contained 12.5 μl of SYBR Green SuperMix (with UDG and ROX, Invitrogen), 0.4 μl each of 10 μM NfV-1-specific primers (p1438, p1439), 3 mM MgCl_2_, 5 μl of the cDNA synthesis reaction, and 10.7 μl of DEPC-water. QPCR conditions consisted of one cycle at 50 °C for 2 min and 95 °C for 10 min, followed by 35 cycles at 95 °C for 15 sec, 60 °C for 1 min. The non-template control for QPCR included a complete cDNA synthesis reaction devoid of RNA template. A standard curve was constructed from a plasmid clone of the corresponding NfV-1 genome region (nucleotides 1011–1187) using a copy number range of 10^3^–10^8^ copies. Reaction efficiencies were determined by regressing *C*_T_ values against the template copy number (log) and calculated according to the formula [*E*=(10^−1/slope^)−1] (Klein et al., 1999). Reaction efficiencies routinely exceeded 90%. Three NfV-1-infected colonies were used for this study. Only individuals in which the virus was detected were included in the analysis (n=41 larvae; 5 pupae; 16 queens; 6 workers).

Active virus replication was evaluated by detection of the replicative genome strand of NfV-1. Total RNA was extracted from a pooled group of workers or larvae (*n*=10) from known NfV-1-infected colonies and used as template for RT-PCR detection of the replicative genome strand by the modified method of Craggs et al. (2001). Total RNA (50 ng) was mixed with 10 mM dNTPs, 1 µM of tagged reverse oligonucleotide primer p1363T (genome region 6051–6080) and heated to 65 °C for 5 min. First strand buffer and Superscript reverse transcriptase (Invitrogen) were then added and the reaction mixture was incubated at 55 °C for 1 h before inactivating the RT at 70 °C for 15 min. Unincorporated cDNA oligonucleotides were digested with 10 units of Exonuclease I (New England Biolabs, Ipswich, MA) at 37 °C for 1 h. The reaction was terminated by heating to 80 °C for 20 min. PCR was subsequently conducted with minus-strand specific cDNA as template. The reaction was conducted in a 25 μl volume containing 2 mM MgCl_2_, 200 μM dNTP mix, 0.5 units of Platinum Taq DNA polymerase (Invitrogen), 0.2 μM of each oligonucleotide primer (p1362) [genome region 6469–6492] and TAG [5′GGCCGTCATGGTGGCGAATAA], and 5 μl of the cDNA preparation. The temperature cycling program was 1 cycle at 94° C for 2 min, 35 cycles of 94 °C for 15 s, 59 °C for 15 s, 68 °C for 30 s, and 1 cycle of 68 °C for 5 min. PCR products were separated on an agarose gel (1%) and visualized by SYBR-safe (Invitrogen) staining.

Host specificity of NfV-1 was examined by testing 9 species of ants within the genus *Nylanderia* (following Kallal and Lapolla, 2012) and two species from the closely related genus *Prenolepis* (LaPolla et al., 2010) for the presence of the virus by RT-PCR (Table 3). These ants were archived samples stored in ethanol. Total RNA was purified from workers from each sample and used as template for reverse transcription and subsequent PCR analysis.

### 4.6 NfV-1 transmission in *Nylanderia fulva*

Horizontal transmission of NfV-1 was evaluated by feeding *N. fulva* colonies a sucrose solution containing a homogenate of NfV-1-infected ants (workers and brood). Eight *N. fulva* laboratory colonies were established from field colonies collected near Winter Garden (Lake Co.) and Callahan (Nassau Co.) Florida. All colonies were determined to be free of NfV-1 infection by RT-PCR. Colonies consisted of an average (±SD) of 1600 (±346) adult worker ants, 1.0 (±1.0) ml of brood (mixture of eggs, larvae, and pupae), and an average of 7 queens (range, 4–10) and were confined to trays with harborage and nesting tubes. Four colonies were starved for 1 day then exposed to virus by feeding them a homogenate of NfV-1-infected ants in 25% (w/v) sucrose solution. A small glass test tube filled with 7–8 ml of the solution and plugged with cotton was placed within each tray. In addition a droplet (1 ml) of the solution was provided on a small weigh boat to provided unfettered access to the virus solution. The ant colonies were allowed to feed on the virus preparation for 3 days and then it was removed and replaced with unadulterated 25% sucrose, water, live house fly (*Musca domestica*) larvae and frozen crickets (*Acheta domesticus*), ad libitum. Four corresponding colonies were used as negative controls and fed 25% sucrose solution without NfV-1-infected ants. Colonies were maintained at ambient room temperature and relative humidity that averaged (±SD) 24.4 (±2.1) °C and 39 (±12.5)%, respectively.

Larval ants (*n*=5 pooled) from each colony were examined for the presence of NfV-1 by extracting total RNA and conducting RT-PCR 6 days after exposure to the virus preparation. Colonies were re-inoculated 16 days after the initial inoculation. Larvae and adult workers (n=5 per colony for each stage) were subsequently sampled at 27, 76, 139 days after the initial inoculation and tested for NfV-1. The number of ants, brood volume, and the presence of queens were recorded for each colony to determine potential colony level effects of virus infection at 2, 10 and 19 weeks after initial inoculation. The percent change in brood volume and worker counts after 19 weeks were compared between persistently infected (NfV-1 detected on more than one sampling) and uninfected colonies by *t*-test (Proc *T* TEST, SAS 9.4, 2012).

Vertical transmission of NfV-1 was evaluated by confining individual queens in 1.7 ml microcentrifuge tubes for 24 h (*n*=19 queens) at 24.9 (±0.9 SD) °C and 23.5 (±0.1 SD)% RH. Eggs were counted within each tube under a dissecting microscope. Queens were obtained from seven colonies used in the transmission test of which NfV-1 was detected in two colonies. A total of 9 queens were sampled from the infected colonies and 2 queens each were collected from the five uninfected colonies. The queens were removed from the vials and placed into a different clean 1.5 ml tubes. The egg clusters were washed with 1 ml of RNAse-free water two times and collected at the bottom of each tube by centrifugation. Total RNA was extracted from the paired queen and water-rinsed eggs and RT-PCR was conducted to detect the presence of NfV-1 as described.

### 4.7 Statistical analysis

The intra-colonial infection rate and stage infection (QPCR) data were subjected to analysis of variance using the general linear models procedure (SAS, 1988). When significant differences were identified by ANOVA, means were separated by Duncan׳s multiple range test. The number of eggs oviposited from infected and uninfected queens were compared by Student׳s *t*-test.

## Figures and Tables

**Table 1:** Strategy used to acquire the genome of NfV-1. Contig 3776.C1 was used as the initial template to design gene specific oligonucleotide primers. From this template, successive 5′ and 3′ RACE reactions were conducted. The regions acquired, amplicon size, and oligonucleotide primers used for cDNA synthesis, and PCR amplification are indicated.

Reaction amplification	Region acquired(nts, 5′→3′)	Size (nts)	Oligonucleotide primers for	
cDNA synthesis	PCR
Contig 3776.C1	5806–7134	1329	N/A	N/A
5′ RACE 1	3189–6538	3350	p1303	p1305/AAP
5′ RACE 2	1257–3500	2244	p1321	p1322/AAP
5′ RACE 3	1–1333	1333	p1168	p1338/AAP
3′ RACE	7001–10881+polyA	3881	oligo dT	p1343/3′ primer

**Table 2:** Field prevalence of the Nylanderia fulva virus 1 in *Nylanderia fulva*.

Collection date	Number of colonies	Stage	Infection rate (%)	Collection location
10.iv.2012	14	Brood	86	Gainesville, Florida
20.vii.2012	8	Brood	88	Gainesville, Florida
15.v.2013	10	Mixture	10	Lakewood Ranch, Florida
21.v.2013	15	Mixture	0	Lakewood Ranch, Florida
22.vii.2013	10	Mixture	0	Gainesville, Florida
23.x.2013	10	Worker	90	Callahan, Florida
15.i.2014	8	Mixture	63	Gainesville, Florida
24.vi.2014	16	Worker	81	Gainesville, Florida
28.viii.2014	4	Mixture	50	Gainesville, Florida
10.ix.2014	3	Mixture	100	Winter Garden, Florida
9.xii.2014	10	Mixture	50	Winter Garden, Florida
26.ii.2015	10	Mixture	80	Winter Garden, Florida
3.vi.2015	6	Brood	17	Winter Garden, Florida
10.ix.2015	25	Brood	44	Callahan, Florida
14.ix.2015	6	Mixture	67	Gainesville, Florida
6.x.2015	3	Mixture	67	Gainesville, Florida
December 2013	10	Worker	0	St. Croix, Virgin Islands
**Summary**	***n*****=168**		**Mean infection rate=52±35%**	

**Table 3:** Evaluation of closely related species for the presence of Nylanderia fulva virus 1.

Presence of Collection date and location	Species (number of nests)	NfV-1
South Carolina: Carolina Sandhills NWR; 9.vi.2009	*Nylanderia arenivaga* (1)	Negative
Florida: White City; 5.viii.2009	*Nylanderia bourbonica* (1)	Negative
Grand Cayman: Mastic Trail; 8.iii.2008; Guadeloupe: Parc National; 24.v.2008	*Nylanderia guatemalensis* (2)	Negative
Massachusetts: Myles Standish State Park; 12.vi.2010	*Nylanderia parvula* (1)	Negative
Massachusetts: Myles Standish State Park; 7.v.2011	*Nylanderia parvula* (1)	Negative
Massachusetts: Myles Standish State Park; 2013–2014	*Nylanderia parvula* (53)	Negative
Florida: Gold Head Branch State Park; 26.viii.2006	*Nylanderia phantasma* (1)	Negative
Trinidad; Anguilla; Barbados; St. Lucia; St. Kitts; 2003–2007	*Nylanderia pubens* (9)	Negative
Illinois: Dixon Springs; 16.iii.2010	*Nylanderia querna* (1)	Negative

Florida: Torreya State Park; 3.x.2008;		

St. Lucia: Savannes Bay; 10.vii.2006;		
Guadeloupe: Mome Cezanne; 4.vi.2011	*Nylanderia steinheili* (3)	Negative
Arizona: Tempe; 11.x.2006	*Nylanderia vividula* (1)	Negative
Florida: Archbold Biological Station; 24.ix.2010	*Nylanderia wojciki* (1)	Negative

Dominican Republic: Parque Nacional Sierra de Bahoruco; 25.vii.2009		
*Nylanderia* n.sp. (1)	Negative
Puerto Rico: Caribbean National Forest; 18.vii.2008	*Nylanderia* n.sp. (1)	Negative
Maryland: Towson University Campus: 17.iii.2011	*Prenolepis imparis* (1)	Negative
Hungary: Balatonfüred; Péterhegy; 31.v.2014	*Prenolepis nitens* (1)	Negative

**Table 4:** Mean percent (±SD) change in the volume of brood (ml) and the number of adult worker ants in NfV-1 infected and uninfected *N. fulva* laboratory colonies 2, 10, and 19 weeks after inoculation with NfV-1 mixed in sucrose solution (25% w/v). Mean (±SD) brood volume and worker counts one day prior to inoculation for the infected colonies (n=3) were 0.5 (±0.58) ml and 1833 (±289) ants, respectively. For the uninfected colonies mean (n=5) brood volume and worker counts were 1.3 (±1.17) ml and 1460 (±321) ants, respectively.

NfV-1	2 weeks		10 weeks		19 weeks	
status	Brood	Worker	Brood	Worker	Brood^a^	Worker^b^
Infected	94 (108)	−11.7 (20.2)	989 (1001)	−8.3 (14.4)	1322 (1368)	15.8 (89.1)
Uninfected	67 (28)	19.6 (34.3)	498 (593)	8.9 (76.4)	644 (1033)	−3.4 (65.1)

a Percent change in brood volume was not significantly different between infected and uninfected colonies (*t*=−0.80; df=6; P=0.4522).

b Percent change in worker numbers was not significantly different between infected and uninfected colonies (*t*=−0.36; df=6; P=0.7341).

**Table 5:** Nylanderia fulva virus 1-specific oligonucleotide primers.

Oligonucleotide designation	Oligonucleotide (5′→3′)	Genome position
p1167	ACCCTACTGACTGACGAACAGATTGCTTC	6816−6847
p1168	TGTTGTTGAGCGTAATGAGTCCGTCCT	6230–6256
p1303	GTCTTGTCAAACGTTTATAATCTATGGCAAATACGTGA	6549–6586
p1305	TCAGCTCCACAAACATGTCATGAAAATTCGCATAGG	6503–6538
p1321	GTTATAAACAGTTTCCATGGTGCATCACACTTATC	3466–3500
p1322	ACGGGACTTGACAAATATTCCATAATTAACTGCCT	4502–4536
p1338	ATTGCTCACCCTTGTTGGGAACGTTACCCTT	1303–1333
p1343	AACTTAAACCTTTCGACACTTGGGAAGAAGTA	7001–7032
p1362	AACGGCACAATAGTCATCGTCCCG	6469–6492
p1363T	TGGCCGTCATGGTGGCGAATAATGTCAACCTAATCT-ACGGTAAGATGAGTCT	6051–6080
p1438	AGATGCAAAGGAACTGACAATGACGAACTT	1011–1040
p1439	TTCATTTGAGCAAGATATAATGAGCGTCTATAGTGGGT	1150–1187

## Footnotes

Appendix A Supplementary data associated with this article can be found in the online version at doi:10.1016/j.virol.2016.06.014.
