# Supplementary material for: Isolation and characterization of Nylanderia fulva virus 1, a positive-sense, single-stranded RNA virus infecting the tawny crazy ant, Nylanderia fulva
Source: Virology. 2016 Sep;496:244–54. doi: 10.1016/j.virol.2016.06.014 (PMC4980443; doi:10.1016/j.virol.2016.06.014)
Supplement: Supplementary file 1 — Supplementary material [file mmc1.docx]

**Supplementary Table 1.** Sequences with similarity to APV, SINV-3 or NfV-1.

| **virus/TSA** | **high-**  **throughput sequencing technology** | **length (nt) excl. poly(A)** | **accession number** | **predicted coding regions** | **host phylum, class and order** |
| --- | --- | --- | --- | --- | --- |
| Acyrthosiphon pisum virus |  | 10013 | NC_003780.1 | join(268..8154,8154..9797), 268..8160, 6716..6949, 6968..7255 | Arthropoda, Insecta, Hemiptera |
| *Sitobion avenae* TSA | 454 | 9772 | assembled from GAPL01023589, 3639, 3644, 3647 and 4163 ^a^ | join(267..8096,8096..9739), 267..8102, 6658..6891, 6910..7197 | Arthropoda, Insecta, Hemiptera |
| Rosy apple aphid virus (host = *Dysaphis plantaginea*) |  | 9963 | DQ286292.1 | join(276..8126,8126..9769), 276..8132, 6688..6921, 6940..7227 | Arthropoda, Insecta, Hemiptera |
| Thika virus (host = *Drosophila* spp.) |  | 9108 | NC_027127.1 | 25..8619 | Arthropoda, Insecta, Diptera |
| Kilifi virus (host = *Drosophila* spp.) |  | 8865 | NC_027126.1 | <1..8604 | Arthropoda, Insecta, Diptera |
| *Clavigralla tomentosicollis* TSA | 454 | 10269 | GAJX01000318.1 | 176..10027 | Arthropoda, Insecta, Hemiptera |
| *Eucyclops serrulatus* TSA | Illumina HiSeq | 12026 | GARW01000621.1 | 75..11972 | Arthropoda, Maxillopoda, Cyclopoida |
| *Anurida maritima* TSA | Illumina HiSeq | 9620 | GAUE01003473.1 | 43..9477 | Arthropoda, Collembola, Poduromorpha |
| *Liposcelis bostrychophila* TSA | Illumina HiSeq | 11448 | GAYV02024882.1 ^b^ | 25..11280 | Arthropoda, Insecta, Psocoptera |
| *Leptinotarsa decemlineata* TSA | Illumina HiSeq | 11614 | GEEF01170301.1 | 17..11476 | Arthropoda, Insecta, Coleoptera |
| Kelp fly virus (host = *Chaetocoelopa sydneyensis*) |  | 11034 | NC_007619.1 ^c^ | 344..10654 | Arthropoda, Insecta, Diptera |
| *Diabrotica virgifera* TSA | 454 | 10231 | GBSB01003728.1 | 31..10194 | Arthropoda, Insecta, Coleoptera |
| *Meligethes aeneus* TSA | Illumina HiSeq | 5536 | GAPE01025462.1 | <2..>5536 | Arthropoda, Insecta, Coleoptera |
| Solenopsis invicta virus 3 |  | 10386 | NC_012531.1 | join(92..7831,7831..10263), 92..7834 | Arthropoda, Insecta, Hymenoptera |
| *Monomorium pharaonis* TSA | Illumina HiSeq | 10116 | LA857567.1 ^d^ | join(26..7723,7723..10026), 26..7789, 6708..7019 | Arthropoda, Insecta, Hymenoptera |
| Nylanderia fulva virus 1 |  | 10881 | KX024775 | 7..10851, 7436..7753 | Arthropoda, Insecta, Hymenoptera |
| *Menopon gallinae* TSA | Illumina HiSeq | 9052 | GAWR01006667.1 | <2..4738, 5018..8374 | Arthropoda, Insecta, Phthiraptera |

^a^ Three frame-shift sequencing errors corrected by amino acid alignment to NC_003780.1.

^b^ Possible mis-assembly at 3' end - post-poly(A) sequence deleted.

^c^ Possible mis-assembly (see text).

^d^ LJ469201, a 10046-nt TSA from *Solenopsis invicta*, has 99% nt identity to LA857567.1.
